# Effect of *Melissa officinalis* (Lemon balm) on Sexual Dysfunction in Women: A Double- blind, Randomized, Placebo-controlled Study

**Published:** 2018

**Authors:** Zahra Darvish-Mofrad-Kashani, Elham Emaratkar, Fataneh Hashem-Dabaghian, Fatemeh Emadi, Firoozeh Raisi, Jale Aliasl, Mohammad Kamalinejad, Seyed Abbas Hasheminejad, Tahere Eftekhar, Nafise Zafarghandi

**Affiliations:** a *Department of Traditional medicine, Faculty of Medicine, Shahed University, Tehran, Iran. *; b *Research Institute for Islamic and Complementary Medicine, Iran University of Medical Sciences, Tehran, Iran. *; c *Traditional medicine clinical trial research center, Shahed university, Tehran, Iran.*; d *Roozbeh Psychiatric Hospital, Tehran University of Medical Science (TUMS),Tehran,Iran. *; e *Department of Pharmacognosy, School of Pharmacy Shaheed Beheshti University of Medical.*; f *Department of Gynecology and Obstetrics, Faculty of Medical Science, Tehran University of Medical Sciences, Tehran, Iran.*; g *Department of Obstetrics and Gynecology Medical Science, Shahed University, Tehran, Iran.*

**Keywords:** Hypoactive sexual desire disorder, Persian Medicine, Lemon balm, Libido, *Melissa officinalis*, Sexual dysfunction, Women

## Abstract

Hypoactive sexual desire disorder (HSDD) is the most prevalent female sexual dysfunction (FSD) and its bio-psychosocial multifactorial etiology justifies its multifaceted treatment. In Persian Medicine (PM), the weakness of the main organs (heart, brain and liver) is one of the important causes of lack of sexual desire; hence, their strengthening is a priority during treatment. *Melissa officinalis* is one of the medicinal plants with tonic characteristics for the main organs in PM and was used for treatment in this study. The aim of the present study was to evaluate the efficacy and safety of *M. officinalis* in the improvement of HSDD in women. Eighty nine (89) eligible women suffering from decreased sexual desire were randomly assigned to groups. The participants received medication (500 mg of aqueous extract of *M. officinalis*) or placebo 2 times a day for 4 weeks. Changes in scores of desire, arousal, lubrication, orgasm, satisfaction and pain were evaluated at the end of 4 weeks of treatment using the Female Sexual Function Index (FSFI) questionnaire in the two groups. Forty three participants completed the study. The increase in desire (*P* < 0.001), arousal (*P* < 0.001), lubrication (*P* < 0.005), orgasm (*P* < 0.001), satisfaction (*P* < 0.001), pain (*P* < 0.002) and FSFI total score (*P* < 0.001) in the M. officinalis group was significantly more than that of the placebo group. The willingness to continue treatment was significantly higher in the M. officinalis as compared to the placebo group (*P* < 0.001). M. officinalis may be a safe and effective herbal medicine for the improvement of HSDD in women.

## Introduction

 Female sexual dysfunction (FSD) is a very prevalent health problem which affects 25 to 65% of women (1). The prevalence of FSD has been estimated to be 43% in America (2) and reported to be 31.5% in Iran (1). Hypoactive sexual desire disorder (HSDD) is the most prevalent sexual dysfunction in women of all ages (3) which impairs emotional health, interpersonal communication (4) and quality of life of women (3, 5).

 HSDD in the Diagnostic and Statistical Manual of Mental Disorders, Fourth Edition, Revised (DSM-IV-TR) is defined as persistent or recurrent deficiency (or absence) of sexual fantasies and desire for sexual activity which causes marked distress or interpersonal difficulties that cannot be explained by any other mental disorder (except another sexual dysfunction) or the direct effect of a substance, and there is no better definition for it in general medical disorders (6). Female hypoactive sexual desire disorder and female arousal disorder have been merged into one disorder: female sexual interest/arousal disorder in the DSM 5th Edition (DSM-5) (7).

 Complex and multifaceted etiology of HSDD includes physiological, psychological and sociological factors which justifies its multifaceted intervention (3, 4). Psychotherapy, behavioral and sex therapy in addition to Pharmacotherapy are considered as the recognized interventions in the domain of decreased sexual desire (5). Transdermal testosterone (3), Bupropion (8), Bremelanotide (9), Dehydroepiandrosterone (10) and Flibanserin (5) are some of the drugs used for decreased sexual desire in women, and among these, only Flibanserin (5) has been recently accepted by America Food and Drug Administration (FDA) (11).

 Medicinal plants for traditional medicine are focused on by researchers in different domains of medical science. Many clinical trials have been conducted in this regard to evaluate the effects of these drugs on different domains of FSD; these include study on Ginseng (12), Saffron (13), *Elaeagnus angustifolia* (14) and *Tribulus terrestris* (15). According to Persian Medicine experts (such as Ibn Sina), strong and healthy body is essential for healthy sexual function which is essential for maintaining good health (16).

 The valuable book, Canon of Medicine, was written by Ibn Sina (One of the most prominent Iranian scientists between 9 and 14 AD) (17). Chapter 12 of the third volume of this book is related to sexual health, measures and treatment of its disorders. In this chapter, libido is described as "Bah" (15) which explains the cause of decreased sexual desire, signs and treatment of any of the causes in detail (18). 

 In Persian Medicine sources, weakness of the main organs (heart, brain and live) is one of the leading causes of decreased sexual desire (*Bah*). Accordingly, strengthening the main organs is the main priority of weak sexual desire treatment (18, 19). *Melissa officinalis* is one of the tonic drugs for the main organs in ITM (20). This medicinal herb was mentioned in PM with the name *"Badranjboye*". The history of use of this medicinal plant is ancient Persia and its cultivation ability in different regions of Iran indicates that it is indigenous, available and it has been used for thousands of years in Iran (21).

*M. officinalis* or lemon balm (Labiate) is an aromatic and slightly spicy plant with lemon aroma and taste. The main components of the essential oil of *M. officinalis* are: geranial (citral a), neral (citral b) and citronellal. Phenolic acids (like rosmarinic acid) are the main components of *M. officinalis* aqueous extract which are mainly water-soluble (22, 23).

 Recent animal and human studies have shown the different therapeutic applications of *M. officinalis* which are: as anti-anxiety (24), antidepressants (25, 26), for improvement of cognitive function and mood (27), for calming and positive effect on the immune system and stress (28), for Alzheimer's disease (29), nervous sleeping disorders and functional gastrointestinal complaints (22). Even though the treatment of nervous debility has been reported to be among the pharmacological effects of *M. officinalis* in PDR which is used in folk medicine (23), there has not been any trial that evaluated its effect on the improvement of decreased sexual desire in women. 

 The objective of this study was to evaluate the effect of *M. officinalis* extract on the improvement of sexual dysfunction in women aged 18 to 50 years. 

## Materials and methods

*Study design*

This was a randomized, double-blind, placebo-controlled study. The Ethics Committee of Shahed University approved the protocol (approval number: 41/195148). In addition, it was registered in the Iranian Registry of Clinical Trials (Registration number: IRCT 2014021516280N1).

The study was conducted in three centers: Shahid Mostafa Khomeini Hospital, Imam Khomeini Hospital and Salamatgah of Iranian Traditional Medicine- Shahed University, from July 2014 to October 2015.

*Study population*

The participants that qualified for the study were selected among 18 to 50 years old women referred to the mentioned centers with complain of decreased sexual desire. They were interviewed face-to-face in order to ensure the occurrence of decreased sexual desire in them. The whole process of interview, examination and history taking was done by traditional medicine assistant that was trained under the supervision of a gynecologist and psychiatrist. The interview was formed based on DSM-IV-TR criteria for HSDD (6).

The participants entered into the study after history taking, examination and completing the FSFI questionnaire (30) and if they met the inclusion criteria.

*Inclusion criteria*: this included women aged 18 to 50 years with satisfying emotional relationship with husband and sexually active at least for one year and having sexual intercourse at least once a month, decreased sexual desire (with a score equal to or less than the cut-off point of sexual desire domain (3.3) in the FSFI questionnaire), lack of pregnancy and lactation, lack of retard menstrual for 2 months or more and normal pelvic exam.

*Exclusion criteria* **: ** this included losing emotionally satisfying relationship with husband, the addition of another sexual partner during the study, the presence of any organic, anatomical or hormonal disease in the study group, such as: diabetes, cerebrovascular disease, damaged liver and kidney function, heart disease, hypothyroidism, history of cancers and psychiatric disorders under medication therapy, smoking, using alcohol, drug abuse and taking any chemical or herbal drug which has effect on sexual desire. 

Details about the project and its purpose were provided by the researcher. Thereafter, the participants completed the consent form before enrolment into the study. 

Participants in both groups had the right to withdraw from the project at any time.

*Medicine Preparation*

Dried leaves of *M. officinalis* were purchased from the Firuze botanical Garden at Shahriar, Tehran, Iran.

The plant was authenticated and deposited at Shahid Beheshti University of Medical Sciences (SBUM) herbarium with voucher no. 8022- sbum.

Preparation of the plant extract was done at Traditional Medicine Department, Faculty of Medicine, Shahed University. 

An aqueous extract of the dried leaves of *M. officinalis* in boiling water was prepared. The extract was concentrated and then filled in 500 mg capsules. The placebo was prepared from starch.

The medication was provided in identical 500 mg capsules, containing either M. officinalis extract or placebo. The participants received either *M. officinalis* extract or placebo, 2 times a day, each time 2 capsules an hour after breakfast and dinner.

*Medicine standardization*

Total phenolic compounds of M. officinalis aqueous extract were determined with Folin–Ciocalteu’s reagent (31) using gallic acid as a standard phenolic compound. The total phenolic content in the extract was 3.32±0.02 mg GA/g. The content of flavonoids in the extract was measured with spectrophotometric method (32) using rutin as a standard. The total flavonoids content in the examined extract was 1.8±0.003 mg RU/g.

*Interventions*

 The participants were randomly divided into two groups: the *M. officinalis* and placebo groups. Randomization was performed using block randomization and blocks of four at a ratio of 1:1 which were assigned to both groups were used.

 Participants received *M. officinalis* extract or placebo according to the code provided by Traditional Pharmaceutical Group of Shahed University and those based on the same guidelines were used.

 Participants and researcher were unaware of the medications included in the box (a double-blind randomized clinical trial).

 Pharmaceutical capsules were packed in 2 containers. The first container was given to the participants at this stage and the second one was given to them after 2 weeks. The shape of capsules and containers was the same in both groups.

 Physical and gynecological examinations together with demographic and base data and medical history collection were done at the beginning of the study. Also, frequency of intercourse in a month was asked and the participants were asked to fill FSFI questionnaire.

 Follow-up on the correct use of the medicine by participants was done by researcher using phone calls after 48 to 72 h. Participants completed the side effects form at the end of the second week at the time of receiving the second package of the medicine. FSFI questionnaire and side effects questionnaire were filled at the end of the fourth week. Changes in the frequency of intercourse in a month were also recorded.

 The participants were asked the following questions in order to evaluate the effectiveness of the treatment: Has the distress caused by sexual dysfunction been improved? and the global efficacy question (GEQ: “Did the treatment you received improve meaningful your sexual desire?”), and overall participant satisfaction question (“Are you satisfied with the efficacy of your treatment?”).

 They were also asked to state their willingness to continue the treatment after the end of the study (8). The questionnaire for participants who used at least 80% of drugs was used for extraction of results.

*Outcomes*

 Participants answered FSFI questionnaire based on their sexual function during the 4 weeks (at the beginning of the study and the end of the fourth week). Self-reported Female Sexual Function Index (FSFI) questionnaire had 19 questions which evaluated the sexual function of women in 6 domains (desire, arousal, lubrication, orgasm, satisfaction and pain).

 Each domain has different coefficient and questions. Questions of each domain are different: sexual desire (questions 1 and 2), arousal (3-6), lubrication (7-10), orgasm (11-13), satisfaction (14-16) and pain (17-19).Scores of each domain were obtained from the sum of scores of the questions in each domain multiplied by the coefficient of that domain. Higher scores indicate the better sexual function in each domain. The questions have five answers and the range of scores is from 1 to 5. The coefficient of desire is 0.6. The score of this domain is variable, between 1.2 and 6. 

 The Cut-off point was defined for each domain and lower point of the cut-off point indicates poor function in that domain. Sexual desire which is the primary outcome of the study and secondary outcomes such as arousal, lubrication, orgasm, satisfaction and pain questionnaire’s total score were compared in both groups by FSFI.

 Comparison of the frequency of intercourse in a month was done between the two groups before and after treatment. The evaluation of the improvement of distress caused by sexual dysfunction was done at the end of the fourth week by obtaining a yes or no answer from the participants. The answer to the GEQ which is: did the treatment you received improve meaningful, your sexual desire? and overall participant satisfaction question which is: are you satisfied with the efficacy of your treatment? had five options including very high, high moderate, low, very low or none. Finally, the participants were asked to express their willingness to continue the treatment after the end of the study by answering yes or no. The side effects were recorded in the second and fourth weeks using the side effects’ check list.

*Sample size & statistical analysis*

 The formula for comparing two means was used to calculate the sample size which was 38 individuals (19 for each group) by considering the type I error of 0.05, study power of 80% and 12.5 standard deviation observed in the pilot study and for at least 10 points difference in the drug group as compared to the placebo (one-way).

 Description of quantitative data was done using mean and standard deviation and description of qualitative data was done using frequency percentage.

 P value lower than 0.05 was considered to be statistically significant.

 Comparison of quantitative variables between the groups was carried out using student t-test and Mann-Whitney U test. Chi square test was used to compare the qualitative variables between the two groups.

## Results

*Participants*

**Figure 1:**
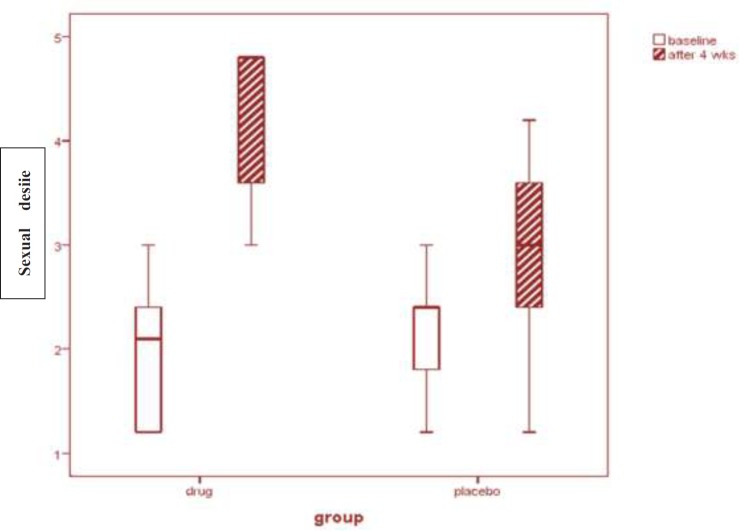
Flow diagram of recruited patients

Initially, 149 participants complaining of decreased sexual desire were interviewed and 89 of them that were eligible were randomly selected: 45 and 44 in the *M. officinalis* and placebo groups, respectively (Figure 1).

Sixty of them were excluded before randomization among which, 34 did not meet the inclusion criteria, 16 met the exclusion criteria and 10 refused to sign a consent. From the 58 participants that received treatment (30 participants that received *M. officinalis *extract and 28 that received placebo), 7 discontinued the intervention (2 in *M. officinalis* group and 5 in placebo group) and 8 missed the follow-up (6 in *M. officinalis* group and 2 in placebo group). Among the 7 participants who discontinued the intervention, 2 were afraid of using *M. officinalis*, 2 were afraid of receiving Placebo, 1 had family disputes and 2 had personal reasons.

The basic characteristics of the study participants are presented in Table 1. There was no significant difference between basic characteristics of these two groups. Mean (± SD) age was 35.5 (7.2) and body mass index (BMI) was 24.8 (3.4).There was no significant difference in the two groups at the beginning of the study (P = 0.960) with regards to sexual intercourse in a month. Forty three participants (22 in *M. officinalis* group and 21 in the placebo group) completed the study.

*Analysis of the outcomes*

**Figure 2:**
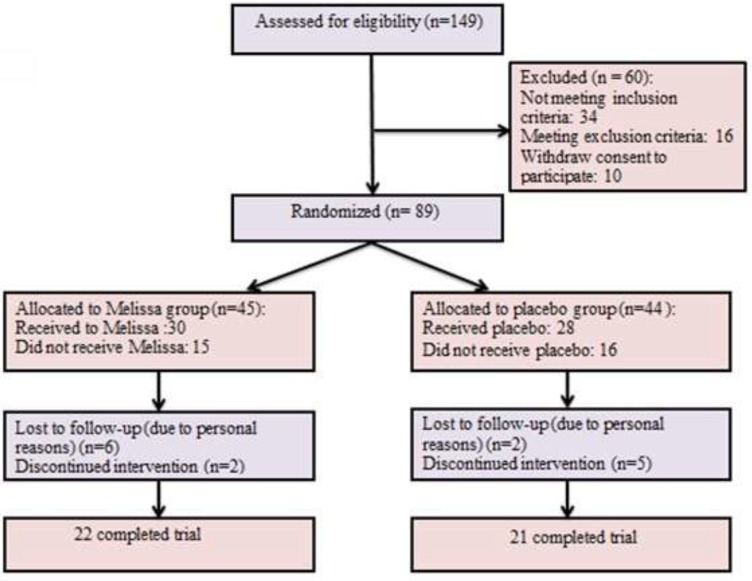
Sexual desire scores before and after the intervention

Desire, the first outcome of the study, was evaluated by the FSFI questionnaire at the baseline and 4 weeks after starting the treatment. The base scores of desire did not show significant difference between the two groups (*P* = 0.361) (Table 2). The results showed a significant increase in the scores of desire in the *M. officinalis* group as compared to the Placebo group after the end of the 4th week of the treatment (*P* = 0.001) (Figure 2).

Arousal was another variable of the study, evaluation of its scores by the FSFI questionnaire indicated that its base scores did not show significant difference between the two groups (*P* = 0.439) while a significant difference was observed in arousal between the *M. officinalis* and Placebo groups after the intervention (*P* = 0.001) (Table 2).

The base scores of lubrication did not show significant difference between the two groups 

(*P* = 0.542). The mean (SD) of this domain increased from 3.21 (1.3) and 3.44 (1.3) at the baseline to 4.93 (0.75) and 3.90 (1.2), respectively in the *M. officinalis* and placebo groups according to FSFI questionnaire which show a significant increase in the results of the *M. officinalis* group as compared to the placebo group (*P* = 0.005) (Table 2).

The base scores of orgasm did not show significant difference between the two groups (P = 0.932). The changes in orgasm from the baseline till the end of the treatment were significant between the *M. officinalis* and placebo groups (*P* = 0.001) (Table 2).

The scores of satisfaction were also evaluated by FSFI questionnaire at baseline and after the intervention. Similar to other domains, there was no significant difference between the two groups at the baseline (*P* = 0.542). Increased scores of the *M. officinalis* after intervention was significant as compared to the placebo group (*P* = 0.001) (Table 2).

Another variable evaluated by FSFI questionnaire was pain. The base scores of pain did not show significant difference between the *M. officinalis* and Placebo groups (*P* = 0.732). The results after completion of treatment showed significant improvement in pain in the *M. officinalis* group as compared to the placebo group (*P* = 0.002) (Table 2).

There was no significant difference between the two groups at the baseline (*P* = 0.519) with regards to the total score of FSFI. The mean (SD) of this domain increased form 18.5 (4.2) and 19.4 (3.9) at the baseline to 27.9 (0.6) and 21.7 (3.9), respectively in the *M. officinalis* and placebo groups according to the FSFI questionnaire which showed a significant increase in the results of the *M. officinalis* group as compared to the placebo group (*P* = 0.001) (Table 2).

The mean (SD) frequency of sexual intercourse in a month showed a significant increase of 9.0 (3.4) in the *M. officinalis* group as compared to 4.3 (2.3) in the placebo group, at the end of the study (*P* = 0.001).

**Figure 3:**
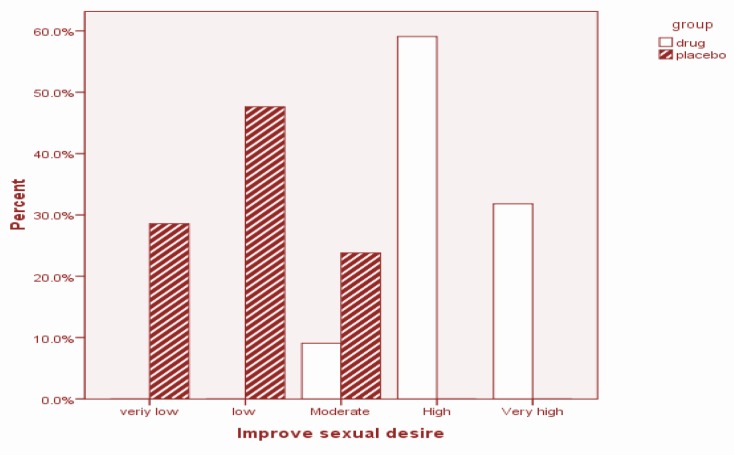
Summary of the participants' response to the GEQ; ‘Did the treatment you received during the 4 weeks improve meaningfully your sexual desire

**Figure 4:**
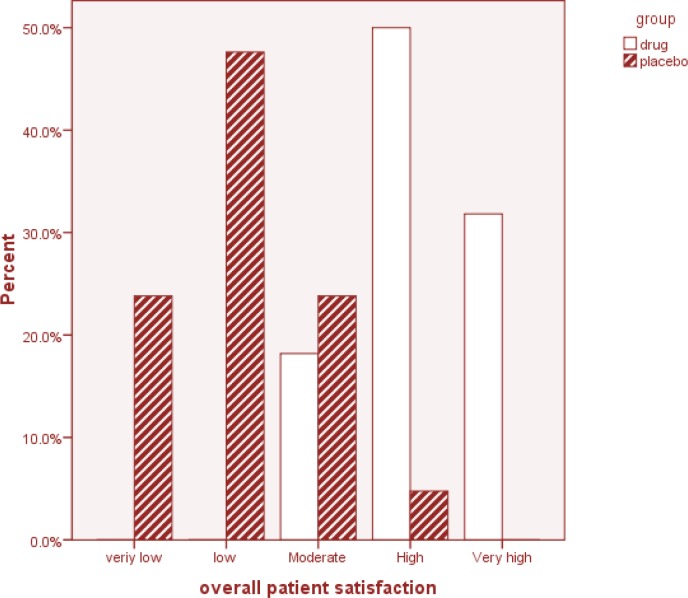
Summary of the participants’ response to the overall patient satisfaction question ‘Are you satisfied with the efficacy of your treatment?’

The participants were asked the GEQ: “Did the treatment you received improve meaningful, your sexual desire?” The answer of the *M. officinalis* group showed a significant improvement after 4 weeks of treatment (*P* = 0.001) (Figure 3). The overall participant satisfaction question: “Are you satisfied with the efficacy of your treatment?” was also asked. The answer given by 7 participants (31.8%) in the *M. officinalis* group was “very high”, while none of the participants in the placebo group gave this answer. With regards to this question, 11 (50%) participants in the *M. officinalis* group and 1 (4.8%) in the Placebo group gave a “high” answer. In general, the rate of overall satisfaction with treatment at the end of the study was significantly higher in the *M. officinalis *group as compared to the Placebo group (*P* = 0.001) (Figure 4). 

The participants were asked about their willingness to continue treatment. 20 (90.9%) in the *M. officinalis* group and 4 (19.0%) in the Placebo group responded positively and those in the *M. officinalis *group were significantly more willing to continue treatment as compared to those in the placebo group (p = 0.001). 

Finally, 21 participants (95.5%) in the *M. officinalis* group and 6 (28.6%) in the Placebo group answered yes to the question: “Has the distress caused by sexual dysfunction been improved after 4 weeks of treatment?” There was a significant differences between the *M. officinalis* and placebo groups (*P* = 0.001) with regards to distress improvement, after the intervention.

*Side effects*

No serious side effect was reported in both groups during the study. Five side effects including nausea, diarrhea, constipation, increased vaginal discharge and vaginal bleeding were observed during the study period and in total, 2 participants (9.1%) in the *M. officinalis* group and 5 (23.8%) in the placebo group had side effects. The Side effects observed with *M. officinalis* extract were diarrhea (4.5%) and constipation (4.5%). The difference between the two groups in terms of side effects was not significant (*P* = .24).

## Discussion

FSD is a very prevalent health problem and HSDD is the most prevalent sexual dysfunction in women of all ages which impairs emotional health, interpersonal communication (4) and quality of life of women (3, 5).

The present study showed improvement of the hypoactive sexual desire disorder by *M. officinalis* extract and the other domains of sexual dysfunction: arousal, lubrication, orgasm, satisfaction and pain, were improved using *M. officinalis* as well.

According to the opinions of Persian Medicine philosophers, the weakness of the main organs is an important cause of decreased sexual desire which makes their strengthening a priority during treatment (18, 19). *M. officinalis* boost multilaterally, the main organs in PM sources including Canon (33) and is useful in strengthening the heart, brain (18) and also the liver (20). *M. officinalis* was used in this study as a tonic for the main organs in the treatment of loss of sexual desire and it may have improved this disorder by strengthening the main organs; this finding confirm this hypothesis. The results of the present study support the unproven effects of *M. officinalis* on PDR for the improvement of nervous debility (23).

**Table 1:** Baseline characteristics of study subjects.

**characteristic**	***Melissa officinalis*** **group (n = 22)**	**Placebo group (n = 21)**	***P*** ** value**
Age (years), mean ± SD Patient	35.2 (7.9)	35.4(6.7)	.893
Partner	42.5(9.7)	41.6(6.5)	.817
Body mass index (Kg/m2), mean ± SD	24.4 (2.9)	25.2(3.9)	.913
Duration of marriage (years), mean ± SD Occupational status: Housewife, n (%) Employed, n (%)	2.9(8.8)	3.9(7.9)	.855
	15(51.7%) 7(50.0%)	14(48.3%) 7(50.0%)	.916
Frequency of sexual activity (Baseline), mean ± SD	4.4 (2.75)	4.1 (.42)	.960

**Table 2:** Changes in FSFI domains scores from baseline to the end of 4 weeks of treatment

**Domains ** **)** **mean ± SD** **(**	**groups**	**Baseline**	* ***P*** ** value**	**After 4 weeks**	* ***P*** ** value**	**intragroup Changes**	* ***P*** ** value**
Desire	M. officinalis	1.99 (.65)	0.36	4.28).62(	0.001	2.2(0.4)	<0.001
	placebo	2.17 (.67)		2.80).85 (		0.6(0.4)	
Arousal	M. officinalis	2.69 (.90)	0.43	4.51(.63)	0.001	1.8(0.9)	<0.001
	placebo	2.88(.95)		3.27(.93)		0.3(0.8)	
Lubrication	M. officinalis	3.21 (1.3)	0.54	4.93(.75)	0.005	1.7(0.4)	0.001
	placebo	3.44(1.3)		3.90(1.2)		0.4(1.2)	
Orgasm	M. officinalis	2.96 (1.2)	0.93	4.87(.80)	0.001	1.9(0.8)	<0.001
	placebo	2.97(.96)		3.23(.94)		0.2(0.7)	
Satisfaction	M. officinalis	3.30 (.86)	0.52	5.18(.77)	0.001	1.8(0.6)	<0.001
	placebo	3.52(.93)		4.00(.80)		0.4(0.8)	
Pain	M. officinalis	4.50 (1.1)	0.73	5.56).65(	0.002	1.06(1)	0.002
	placebo	4.48(.99)		4.78).85 (		0.2(0.5)	
Total FSFI score	M. officinalis	18.5 (4.2)	0.51	27.9(6.0)	0.001	9.3(6.6)	<0.001
	placebo	19.4 (3.9)		21.7(3.9)		2.2(4)	

* P value for comparison between groups.

The demographic and baseline characteristics of two groups of participants were similar. Thus, the improvement of sexual desire can be a result of *M. officinalis* extract due to its strengthening effects on the main organs in the participants.

According to the present survey, there is no other study on the effect of *M. officinalis* on sexual desire. The study evaluated the effect of *M. officinalis* extract on hypoactive sexual desire disorder using a double-blind randomized clinical trial for the first time.

Kennedy *et al.* (2006) and EmamGhoreishi and Talebianpoor (2009), respectively reported the anti-anxiety and antidepressant effects of *M. officinalis* (24, 25); the *M. officinalis* significantly improved the desire, arousal, orgasm and satisfaction of FSFI which can be due to its anti-depressant and anti-anxiety effects. It seems that this point can be in line with opinions on ITM about strengthening the brain and heart by this plant which has also been mentioned in the Canon book (18, 20). Evaluation of the mechanism of *M. officinalis* in the study of EmamGhoreishi showed that *M. officinalis* aqueous extract probably had antidepressant effects by increasing norepinephrine (NE) similar to imipramine (22). Since NE plays a catalytic role in sexual desire (34, 35), the effect of *M. officinalis *on this domain may be related to this mechanism of action.

The effect of *M. officinalis* on improvement of cognitive performance and mood was reported by Kennedy *et al*. (27). Akhondzadeh *et al*. also used *M. officinalis* extract in the treatment of patients with mild to moderate Alzheimerꞌs disease. The studies showed that the mechanism of action of *M. officinalis* on the mentioned domain may be based on cholinergic activity and stimulation of CNS acetylcholine. The evaluation of the mechanism of *M. officinalis* aqueous extract on isolated rat heart in the study of Gazola *et al*. showed that the muscarinic receptor stimulation may lead to reduced heart rate without changes in contractions power (36). *M. officinalis* significantly increased the score of sexual arousal in the present study. According to studies, the parasympathetic nerve stimulation and release of acetylcholine are affective in the creation of the physical changes that occur during sexual arousal (including genital vascular vasodilatation) (33, 35). Even though the mechanisms of action of *M. officinalis* on arousal of women is unknown and requires further researches, these genital changes caused by sexual arousal may be caused by the effect of *M. officinalis* on muscarinic receptors.

Ballard *et al.* showed the effect of *M. officinalis* on the treatment of agitation in severe dementia (37). Antimicrobial and antioxidant activities of *M. officinalis* were reported in 2004. The results of the current study are not in line with the findings of the studies mentioned.

Dopamine has a stimulatory effect on desire and subjective arousal and facilitates sexual behavior while prolactin plays an inhibitory role in this domain (34, 35, and 39). The study of Sourgens *et al*. showed that *M. officinalis* reduces the pituitary reserves and serum prolactin level which may be related to increased dopamine in the hypothalamus (26). The improvement of sexual desire observed in the current study can partly be related to the effect of *M. officinalis* on the above neurotransmitter-hormone system. However, more detailed studies are necessary to evaluate this mechanism.

The analgesic effect of *M. officinalis* has been reported in several studies (40, 41). It was shown in the study of Gorji *et al*. that the analgesic effect of *M. officinalis* aqueous extract may be due to its effect on opioid receptors; it was also observed that Naloxone reduces the analgesic effect of extract (40). The beneficial effect of *M. officinalis* on opioid withdrawal syndrome was reported in a review study of the effect of Iranian herbal medicines on opioid withdrawal syndrome (42). It was observed in the study of Wilson *et al.* in 2009 that opioids and neuropeptides can be effective on sexual pain disorder (43). Since the *M. officinalis* significantly improved pain domain score in this study, this effect may be due to action on opioid system.

No serious complication was observed in the present study and there was no significant difference between intervention and placebo groups in terms of side effects which indicates the safety of *M. officinalis* in the present study*. M. officinalis* is in the list of safe drugs in FDA (26) which is confirmed by the results of this study. This result is in line with safety information of *M. officinalis *in PDR and Commission E (22, 23) and supports safety findings of *M. officinalis* in other studies (27, 29, 33 and 37). Most drugs used to treat loss of sexual desire have side effects which prevents their effective use in treatment, among which are the potential side effects of androgens replacement therapy including acne, hirsutism, male-building, changes of the voice, erythrocytosis, hepatotoxicity, plasma lipid changes, etc. (44). Side effects such as acne and hirsutism which occur during the systemic administration of DHEA can be listed as examples (38). So, it seems that *M. officinalis* aqueous extract can be an effective and safe treatment for hypoactive sexual desire disorder. 

It is shown that *M. officinalis* is effective in the treatment of sexual dysfunction in women but privacy of sexual problems together with prudency arising from religion and culture are obstacles for participants to talk about sexual problems and may be the reason for difficult surveillance and the main reason for participants leaving the study. It is suggested that *M. officinalis* should be used in larger samples, different statistical populations and longer studies. 

## Conclusions

*M. officinalis* aqueous extract improved desire in women with hypoactive sexual desire disorder and had beneficial effects on sexual arousal, lubrication, orgasm, satisfaction and pain domains of the sexual dysfunction in women and may be used as an effective and safe therapy in female sexual dysfunction.

## References

[1] Safarinejad M (2006). Female sexual dysfunction in a population-based study in Iran: prevalence and associated risk factors. Int. J. Impot. Res.

[2] Shifren JL, Monz BU, Russo PA, Segreti A, Johannes CB (2008). Sexual problems and distress in United States women: prevalence and correlates. Obstet. Gynecol.

[3] Kingsberg SA, Woodard T (2015). Female sexual dysfunction: focus on low desire. Obstet. Gynecol.

[4] Palacios S (2011). Hypoactive Sexual Desire Disorder and current pharmacotherapeutic options in women. Womens Health.

[5] Parish SJ, Hahn SR (2016). Hypoactive Sexual Desire Disorder: A Review of Epidemiology, Biopsychology, Diagnosis, and Treatment. Sex. Med. Rev.

[6] Association AP (2000). Diagnostic and statistical manual of mental disorders: DSM-IV-TR®.

[7] Association AP (2013). Diagnostic and statistical manual of mental disorders: DSM-IV-TR®.

[8] Safarinejad MR, Hosseini SY, Asgari MA, Dadkhah F, Taghva A (2010). A randomized, double-blind, placebo-controlled study of the efficacy and safety of bupropion for treating hypoactive sexual desire disorder in ovulating women. BJU International.

[9] Portman DJ, Edelson J, Jordan R, Clayton A, Krychman ML (2014). Bremelanotide for hypoactive sexual desire disorder: analyses from a phase 2B dose-ranging study. Obstet. Gynecol.

[10] Labrie F, Archer D, Bouchard C, Fortier M, Cusan L, Gomez J-L, Girard G, Baron M, Ayotte N, Moreau M (2009). Effect of intravaginal dehydroepiandrosterone (Prasterone) on libido and sexual dysfunction in postmenopausal women. Menopause.

[11] Hayslett RL (2015). Sexual Dysfunction in Women. US Pharm.

[12] Oh K-J, Chae M-J, Lee H-S, Hong H-D, Park K (2010). Effects of Korean Red Ginseng on Sexual Arousal in Menopausal Women: Placebo-Controlled, Double-Blind Crossover Clinical Study. J. Sex. Med.

[13] Kashani L, Raisi F, Saroukhani S, Sohrabi H, Modabbernia A, Nasehi AA, Jamshidi A, Ashrafi M, Mansouri P, Ghaeli P (2013). Saffron for treatment of fluoxetine-induced sexual dysfunction in women: randomized double-blind placebo-controlled study. Hum. Psychopharmacol. Clin. Exp.

[14] Akbarzadeh M, Zeinalzadeh S, Zolghadri J, Mohagheghzadeh A, Faridi P, Sayadi M (2014). Comparison of Elaeagnus angustifolia Extract and Sildenafil Citrate on Female Orgasmic Disorders: A Randomized Clinical Trial. J. Reprod. Infertil.

[15] Akhtari E, Raisi F, Keshavarz M, Hosseini H, Sohrabvand F, Bioos S, Kamalinejad M, Ghobadi A (2014). Tribulus terrestris for treatment of sexual dysfunction in women: randomized double-blind placebo-controlled study. DARU J. Pharm. Sci.

[16] Darvish- Mofrad- Kashani Z, Zafarghandi N, Raisi F, Aliasl J, Mokaberinejad R, Emaratkar E, Emadi F, Eftekhar T (2016). A review of sexual health opinion from the perspective of iranian traditional medicine. Med. History.

[17] Mokaberinejad R, Zafarghandi N, Bioos S, Dabaghian FH, Naseri M, Kamalinejad M, Amin G, Ghobadi A, Tansaz M, Akhbari A (2012). Mentha longifolia syrup in secondary amenorrhea: a double-blind, placebo-controlled, randomized trials. DARU J. Pharm. Sci.

[18] Ibn-e-sina (Avicenna Husain) (2005). Al-Qanun fit-tib (The Canon of Medicine).(research of ebrahim shamsedine).

[19] Jorjani SE (2005). Al- Aghraz al- Tibbia val Mabohess al- Alaiia.

[20] Aghili Khorasani Shirazi SMH (2001). Makhzan al-Advyh.

[21] Soltani A (2012). Encyclopedia of traditional medicine (medicinal plant).

[22] Blumenthal M, Goldberg A, Brinckmann J (2000). Herbal Medicine-Expanded Commission E Mongraphs. Integrative Medicine Communications.

[23] Barnes J, Anderson LA, Phillipson JD, Newall CA (2007). PDR for Herbal medicines.

[24] Kennedy DO, Little W, Haskell CF, Scholey AB (2006). Anxiolytic effects of a combination of Melissa ofcinalis and Valeriana ofcinalis during laboratory induced stress. Phytother. Res.

[25] Emamghoreishi M, Talebianpour MS (2009). Antidepressant effect of Melissa officinalis in the forced swimming test. DARU J. Pharm. Sci.

[26] Ulbricht C, Brendler T, Gruenwald J, Kligler B, Keifer D, Abrams T, Woods J, Boon H, Kirkwood C, Hackman D (2004). Lemon balm (Melissa officinalis L): an evidence-based systematic review by the Natural Standard Research Collaboration. J. Herb. Pharmacother.

[27] Kennedy D, Wake G, Savelev S, Tildesley N, Perry EK, Wesnes KA, Scholey AB (2003). Modulation of mood and cognitive performance following acute administration of single doses of Melissa officinalis (Lemon balm) with human CNS nicotinic and muscarinic receptor-binding properties. Neuropsychopharmacology.

[28] Bağdat RB, Coşge B (2006). The essential oil of lemon balm (Melissa officinalis L) its components and using fields. J. Fac. Agric. OMU.

[29] Akhondzadeh S, Noroozian M, Mohammadi M, Ohadinia S, Jamshidi A, Khani M (2003). Melissa officinalis extract in the treatment of patients with mild to moderate Alzheimer’s disease: a double blind, randomised, placebo controlled trial. J. Neurol. Neurosurg. Psychiatry.

[30] Rosen C, Brown J, Heiman S, Leiblum C, Meston R, Shabsigh D, Ferguson R, DꞌAgostino R (2000). The Female Sexual Function Index (FSFI): a multidimensional self-report instrument for the assessment of female sexual function. J. Sex. Marital Ther.

[31] Marinova D, Ribarova F, Atanassova M (2005). Total phenolics and total flavonoids in Bulgarian fruits and vegetables. J. Univ. Chem. Technol. Metallurgy.

[32] Beketov E, Pakhomov V, Nesterova O (2005). Improved method of flavonoid extraction from bird cherry fruits. Pharm. Chem. J.

[33] Alijaniha F, Naseri M, Afsharypuor S, Fallahi F, Noorbala A, Mosaddegh M, Faghihzadeh S, Sadrai S (2015). Heart palpitation relief with Melissa officinalis leaf extract: Double blind, randomized, placebo controlled trial of efficacy and safety. J. Ethnopharmacol.

[34] Basson R, Danforth DN, Scott j (2008). Womenꞌs Sexuality and Sexual Dysfunction. Danforth’s Obstetrics and Gynecology.

[35] Berek JS (2012). Berek & Novak s Gynecology.

[36] Gazola R, Machado D, Ruggiero C, Singi G, Alexandre MM (2004). Lippia alba, Melissa officinalis and Cymbopogon citratus: effects of the aqueous extracts on the isolated hearts of rats. Pharmacol. Res.

[37] Ballard CG, O Brien JT, Reichelt K, Perry EK (2002). Aromatherapy as a safe and effective treatment for the management of agitation in severe dementia: the results of a double-blind, placebo-controlled trial with Melissa. J. Clin. Psychiatry.

[38] Mimica-Dukic N, Bozin B, Sokovic M, Simin N (2004). Antimicrobial and antioxidant activities of Melissa officinalis L(Lamiaceae) essential oil. J. Agric. Food Chem.

[39] Clayton AH (2010). The pathophysiology of hypoactive sexual desire disorder in women. Int. J. Gynaecol. Obstet.

[40] Miladi Gorji H, Rashidi Pour A, Vafaei AA, Taherian AA (2006). Opioid receptors role on anti-nociceptive effects of the aqueous extracts of Melissa officinalis in mice. J. Hormozgan Univ. Med. Sci.

[41] Guginski G, Luiz AP, Silva MD, Massaro M, Martins DF, Chaves J, Mattos RW, Silveira D, Ferreira VM, Calixto JB (2009). Mechanisms involved in the antinociception caused by ethanolic extract obtained from the leaves of Melissa officinalis (lemon balm) in mice. Pharmacol. Biochem. Behav.

[42] Ebrahimie M, Bahmani M, Shirzad H, Rafieian-Kopaei M, Saki K (2015). A review study on the effect of Iranian herbal medicines on opioid withdrawal syndrome. J. Evid. Based Complementary Altern. Med.

[43] Wilson LA, Wayman CP, Jackson VM (2009). Neuropeptide modulation of a lumbar spinal reflex: Potential implications for female sexual function. J. Sex. Med.

[44] Hollingsworth M, Berman J (2006). The role of androgens in female sexual dysfunction. Sexuality, Reproduction and Menopause.

